# Analysis of the correlation between P53 and Cox-2 expression and prognosis in esophageal cancer

**DOI:** 10.3892/ol.2015.3624

**Published:** 2015-08-19

**Authors:** JUN CHEN, FANG WU, HONG-LEI PEI, WEN-DONG GU, ZHONG-HUA NING, YING-JIE SHAO, JIN HUANG

**Affiliations:** 1 Department of Radiation Oncology, The Third Affiliated Hospital, Soochow University, Changzhou, Jiangsu 213003, P.R. China; 2 Department of Oncology, The Third Affiliated Hospital, Soochow University, Changzhou, Jiangsu 213003, P.R. China

**Keywords:** esophageal cancer, P53, Cox-2, relevance, prognosis

## Abstract

The present study aimed to explore the importance of P53 and Cox-2 protein expression in esophageal cancer and assess their influence on prognosis. The expression of P53 and Cox-2 was assessed in esophageal cancer samples from 195 patients subjected to radical surgery at Changzhou First People's Hospital (Changzhou, China) between May 2010 and December 2011. Expression of P53 and Cox-2 proteins were detected in 60.5% (118/195) and 69.7% (136/195) of the samples, respectively, and were co-expressed in 43.1% (84/195) of the samples. A correlation was identified between P53 expression and overall survival (OS) (P=0.0351) as well as disease-free survival (DFS) (P=0.0307). In addition, the co-expression of P53 and Cox-2 also correlated with OS (P=0.0040) and DFS (P=0.0042). P53 expression (P=0.023), TNM staging (P<0.001) and P53/Cox-2 co-expression (P=0.009) were identified as independent factors affecting OS in patients with esophageal cancer via a Cox multivariate regression model analysis. A similar analysis also identified P53 expression (P=0.020), TNM staging (P<0.001) and P53/Cox-2 co-expression (P=0.008) as independent prognostic factors influencing DFS in these patients. Binary logistic regression analysis demonstrated a correlation between P53 expression (P=0.012), TNM staging (P<0.001), tumor differentiation level (P=0.023) and P53/Cox-2 co-expression (P=0.021), and local recurrence or distant esophageal cancer metastasis. The results of the present study indicate that P53 and Cox-2 proteins may act synergistically in the development of esophageal cancer, and the assessment of P53/Cox-2 co-expression status in esophageal cancer biopsies may become an important diagnostic criterion to evaluate the prognosis of patients with esophageal cancer.

## Introduction

Esophageal cancer is one of the seven leading causes of cancer-related mortality and is highly malignant (1). China has the highest incidence of esophageal cancer worldwide, and more specifically esophageal squamous cell carcinoma, with the mortality rate associated with this cancer ranking fourth among malignant tumors (2). Atypical early symptoms, middle-to-late stage diagnosis, low treatment remission rates and high local recurrence rates all contribute to the poor prognosis of patients with esophageal cancer. The development and incidence of esophageal cancer involves a multi-factor, multi-step and multi-stage process. The necessary strategies to improve the prognosis and survival rates in patients with esophageal cancer require early discovery, diagnosis and treatment, which rely on studying and exploring the factors that influence the prognosis of esophageal cancer.

The P53 gene displays the highest correlation with human types of cancer thus far. The past decade has witnessed three shifts in the understanding of the association between P53 and cancer, starting from P53 as a protein antigen to P53 as a cancer-associated gene, and finally, to P53 as a tumor-suppressor gene (3). This last advancement arose from the identification of an important dominant-negative mutated P53 gene product acting as an oncogene alleviating the normal tumor suppressor function of wild-type P53 (3). The human Cox-2 gene is located on chromosome 1q25.2-q25.3 and participates in the occurrence and development of tumors by promoting cell proliferation, restraining cell apoptosis, promoting angiogenesis and suppressing immune functions (4). The aims of the present study were to assess the P53 and Cox-2 expression levels in esophageal cancer and to analyze the correlation between P53 and Cox-2 co-expression and the prognosis of esophageal cancer.

## Materials and methods

### 

#### Clinical data

Tumor samples from 195 patients (150 men and 45 women, aged 34–83 years, with a median age of 62 years) diagnosed with esophageal cancer and who underwent radical surgery at Changzhou First People's Hospital (Changzhou, China) between May 2010 and December 2011 were studied. The present study was approved by the Institutional Review Board of Soochow University (Changzhou, China), according to the Declaration of Helsinki. Data regarding age, demographics, tumor location, staging, pathology, adjuvant radiotherapy and survival outcomes were obtained with the written informed consent of each patient. All specimens were associated with a definite pathological immunohistochemical report and detailed follow-up and prognosis data. Of these 195 cases, 194 were identified as squamous carcinoma and 1 case as adenosquamous carcinoma. According to the seventh edition of the international TNM staging criteria of esophageal cancer (5): 11 cases were in stage IA; 11, in stage IB; 33, in stage IIA; 58 in stage IIB; 41, in stage IIIA; 15, in stage IIIB; and 26 in stage IIIC.

#### Immunohistochemical analysis

The archived formaldehyde-fixed paraffin-embedded esophageal cancer specimens were serially cut into 4-µm slices and stained using the two-step Envision Immunochemistry kit (Dako, Glostrup, Denmark). Validated breast cancer specimen sections were used as a positive control, and phosphate-buffered saline was used instead of primary antibodies as a negative control. Monoclonal anti-P53 antibody was obtained from Fuzhou Maixin Biotechnology Development Co., Ltd. (Fuzhou, China), and monoclonal anti-Cox-2 antibody was obtained from Beijing Zhongshan Jinqiao Biotechnology Co. Ltd. (Beijing, China).

#### Evaluation standards for results

All staining results were analyzed by two double-blinded pathological evaluations. Tan nuclear staining indicated positive P53 expression, and tan cytoplasmic staining indicated positive Cox-2 expression. Five randomly selected fields were analyzed for a total of 500 scored cells using a Leica DM2500 microscope (Leica Camera AG, Wetzlar, Germany). For unstained cells, a score of 0 was specified. For stained cells, 1–19% of cells indicated weak staining intensity (1 point), 20–49% indicated moderate staining intensity (2 points), and ≥50%, appearing as dark brown staining, indicated strong staining intensity (3 points). The scores were then divided into two groups: Scores of 0 and 1 as the negative expression group (-), and scores of ≥2 points as the positive expression group (+).

#### Follow-up

The 195 patients were followed up over a minimal period of 2 years until December 31, 2013, and the median follow-up time was 30 months (range, 2–43 months). No cases were lost resulting in a follow-up rate of 100.00%.

#### Statistical analysis

The statistical analysis was performed using the SPSS statistical software for Windows version 17.0 (SPSS, Inc., Chicago, IL, USA). Survival curves are presented as Kaplan-Meier curves, and significance was classified by the log-rank test. The Cox regression model was used for multivariate prognostic analysis, and a binary logistic regression model was used for the correlation analyses to analyze the influencing clinical factors.

## Results

### 

#### Mortality rate due to recurrence or metastasis

On December 31, 2013, 144 patients had survived and 51 patients had succumbed to tumor recurrence or metastasis. Of those 51 mortality cases, 15 patients exhibited anastomotic recurrence; 13, regional lymph node recurrence; 7, liver metastasis; 8, lung metastasis; 2, bone metastasis; 3, pleural metastasis; and 3, multi-organ metastasis.

#### Correlation analyses between P53 and Cox-2 expression, P53/Cox-2 co-expression and clinical factors

**Figure 1.**
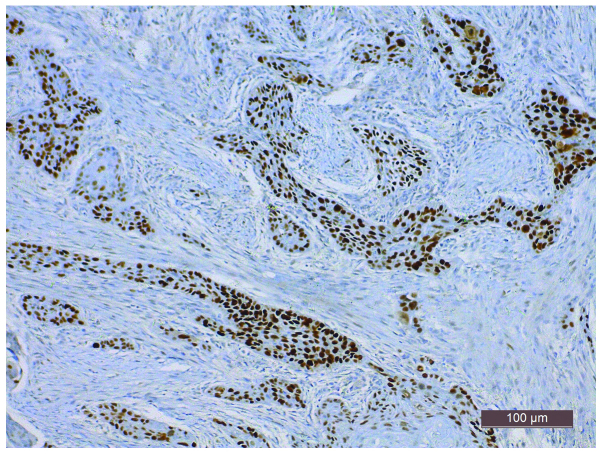
Expression of P53 in the esophagus in esophageal carcinoma [Envision Immunochemistry kit (Dako, Glostrup, Denmark); magnification, x200].

**Figure 2.**
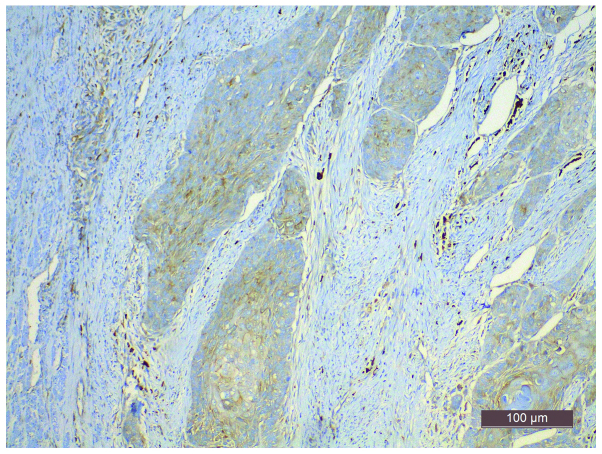
Expression of Cox-2 in the esophagus in esophageal carcinoma [Envision Immunochemistry kit (Dako, Glostrup, Denmark); magnification, x200].

Positive P53 expression, assessed by tan granular staining in tumor cell nuclei, was observed in 60.5% (118/195) of the specimens (Fig. 1). Positive Cox-2 expression, assessed by cytoplasmic yellow staining, was observed in 69.7% (136/195) of the specimens (Fig. 2). In 43.1% (84/195) of the specimens, the co-expression of P53 and Cox-2 was observed, while 17.4% (34/195) of the specimens expressed P53 only and 26.7% (52/195) expressed Cox-2 only. A total of 12.8% (25/195) of the specimens were negative for P53 and Cox-2. P53 expression and P53/Cox-2 co-expression were associated with the age of the patient (P=0.028) and tumor differentiation status (P=0.015; Table I).

#### Correlation analyses between P53 and Cox-2 expression and P53/Cox-2 co-expression, and overall survival (OS) or disease-free survival (DFS)

**Figure 3.**
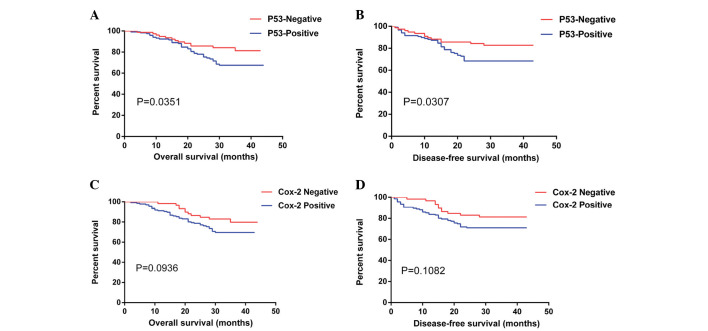
Survival curves of P53 and Cox-2 expression. (A) OS and (B) DFS curves of P53 expression. (C) OS and (D) DFS curves of Cox-2 expression. OS, overall survival; DFS, disease-free survival.

**Figure 4.**
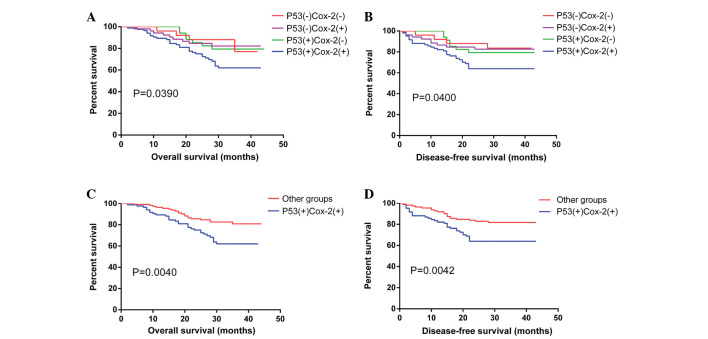
Survival curves of P53/Cox-2 co-expression (A) OS curves of P53/Cox-2 co-expression (B) DFS curves of P53/Cox-2 co-expression (C) OS difference curves of P53/Cox-2 co-expression (D) DFS difference curves of P53/Cox-2 co-expression.

Single factor log-rank analysis by Kaplan-Meier survival analysis were used to assess the association between P53 and Cox-2 expression as well as P53/Cox-2 co-expression and DFS or OS following radical surgery in patients with esophageal cancer. Differences between the OS (χ^2^=4.440, P=0.0351) and DFS (χ^2^=4.672, P=0.0307) curves according to P53 expression were observed, with a two-year OS of 78.0% in the P53-positive group compared with 85.7% in the P53-negative group (Fig. 3). The DFS of the P53-positive group was 68.4% compared with 82.8% for the P53-negative group. No statistically significant differences (P>0.05) were observed for Cox-2 expression in the OS and DFS curves. DFS (χ^2^=8.277, P=0.0040), and OS (χ^2^=8.203, P=0.0042) curves were also affected by the P53/Cox-2 co-expression status, with a two-year OS of 75.0% for the double-positive group compared with 85.6% for the other groups, and a DFS of 63.9% in double-positive patients compared with 82.8% in the other groups (Fig. 4).

#### Relevance of clinical pathological factors with prognosis

Eight risk factors (gender, age, tumor location, TNM stage, tumor differentiation degree, P53 and Cox-2 expression and P53/Cox-2 co-expression) were included in a multifactor analysis using the Cox multivariate regression model with a forced entry method. The results showed that TNM staging [hazard ratio (HR)=3.379, P<0.001], P53 expression (HR=2.102, P=0.023) and P53/Cox-2 co-expression (HR=2.212, P=0.009) were all independent factors affecting the OS curves of patients with esophageal cancer. The same independent prognostic factors also influenced the DFS curves (TNM staging, HR=3.497, P<0.001; P53 expression, HR=2.138, P=0.020; P53/Cox-2 co-expression, HR=2.221, P=0.008) (Table II). The same eight risk factors were also analyzed by the binary logistic regression model with a forced entry method. The results showed that the tumor differentiation degree [odds ratio (OR)=1.964, P=0.023], TNM staging (OR=3.206, P<0.001), P53 expression (OR=2.510, P=0.012) and P53/Cox-2 co-expression (OR=2.204, P=0.021) were associated with the local recurrence or distant metastasis of esophageal cancer (Table III).

## Discussion

P53 is a known tumor-suppressor gene that participates in the occurrence and development of esophageal cancer. The P53 gene is located on human chromosome 17p13 and is composed of 10 exons and 11 introns, encoding a protein 393 amino acids in length. P53 gene products can be divided into wild-type (wtp53) and mutant (mtp53). Upon DNA damage, increased P53 protein expression regulates target genes involved in preventing cells in the G_1_ phase from entering the S phase, which favors DNA repair. If the DNA is seriously damaged, P53 will trigger apoptosis to remove the cells with the overly damaged DNA. Tumor growth requires angiogenesis, and Kang *et al* (6) demonstrated that the P53 gene functions by inhibiting tumor angiogenesis via the adjustment of platelet response protein 1 (TSP-1) levels, which is the main angiogenesis inhibiting factor. However, mtp53 acts as a proto-oncogene by promoting the occurrence and development of tumor cells. Huang *et al* (7) showed that the P53 expression level in normal tissue is only one-eighth of that in tumor tissues; furthermore, since the P53 protein has a short half-life, it can hardly be detected in normal cells. However, when cells become damaged or mutated by various factors, P53 expression increases significantly. Mtp53, instead of inhibiting tumor cell proliferation, promotes cell proliferation and eventually alters the cellular phenotype in a malignant manner (8).

Previous studies have demonstrated that the P53 gene mutation is associated with poor prognosis in various types of cancer, including colon, breast, lung, gastric and esophageal cancer (9,10). Overexpression of P53 in esophageal tumor cells increases their potential to invade tissue and blood vessels, and promotes the local recurrence and metastasis of esophageal cancer, leading the progression towards late pathological staging and poor prognosis (11). In the present study, it was revealed that P53 expression was associated with age and tumor differentiation degree (P<0.05). In patients ≥60 years old, P53 expression was found in 66.1% (84/127) of the cases, and in patients with poorly differentiated cancer, P53 expression was observed in 69.0% (49/71) of the cases. Han *et al* (12) showed that P53 expression was positively correlated with tumor stage and lymph node metastasis. Ye *et al* (13) noted that P53 expression was not associated with the gender or age of the patient, but was associated with tumor differentiation degree and lymph node metastasis. Finally, Chino *et al* (14) showed that P53 expression was not associated with tumor infiltration depth, lymph node metastasis or venous or lymphatic invasion. Such differences in findings between studies may be caused by the different stages and sources of samples, different P53 antibodies or variations in the experimental methods. Jin *et al* (15) used an immunohistochemical method to detect the expression level of P53 in 80 specimens of esophageal carcinoma and different diseased tissues *in situ*, which implied that positive P53 expression was associated with the occurrence and stage of esophageal squamous cell carcinoma and could be used to identify high-risk individuals in a precancerous population. In the present study, single factor Kaplan-Meier analysis showed a difference in OS curves according to P53 expression (χ^2^=4.440, P=0.0351), with a two-year OS of 85.7% in the P53-negative group compared with 78.0% in the P53-positive group. Similarly, P53 expression also influenced the DFS curves (χ^2^=4.672, P=0.0307), with a two-year DFS of 82.8% in the P53-negative group compared with 68.4% in the P53-positive group. In addition, a Cox multivariate regression analysis identified P53 expression as an independent factor affecting patient survival rate, and a binary logistic regression analysis showed that P53 expression was associated with local recurrence or distant metastasis following esophagectomy.

Cox-2 plays a role in the development of esophageal cancer. Prostaglandin-endoperoxide synthase (PTGS), also known as cyclooxygenase, is a monotopic membrane protein which acts as a rate-limiting enzyme for the conversion of arachidonic acid into prostaglandins. The PTGS family comprises Cox-1 and Cox-2, which regulate different cellular functions despite their homology (16). Cox-1 is expressed in the majority of normal tissues, whereas the Cox-2 enzyme is induced rapidly in response to pathological states, such as inflammation and tumor formation (17,18). A previous study has also shown that Cox-1 has an induced type and Cox-2 has a structured type, and that a variant named Cox-3 (an isomer of Cox-1) also possibly exists (19). The human Cox-2 gene, located on chromosome 1q25.2-q25.3, is composed of 9 introns and 10 exons encoding a protein of 604 amino acid residues. In normal tissue, Cox-2 expression is low or absent. Cox-2 expression is induced by various cellular factors, including proinflammatory responses, and is involved in tumor development, invasion and metastasis (20). According to Misra *et al* (21), Cox-2 participates in the occurrence and development of esophageal cancer in multiple ways, including by inhibiting the apoptosis or promoting the proliferation of tumor cells and accelerating invasion and metastasis; however, the specific mechanism remains unclear. Okumura *et al* (22) noted the important role of Cox-2 in the synthesis of prostaglandin and its role in mediating angiogenesis, tumor growth, invasion and metastasis. Kashiwagi *et al* (23) revealed that Cox-2 may increase vascular endothelial growth factor-C expression by generating prostaglandin, thus promoting the generation of lymphatic vessels in tumor tissues and favoring metastasis possibly through the lymph nodes. Zhou *et al* (24) measured Cox-2 expression and lymphatic vessel density (MLD) in esophageal cancer tissues by an immunohistochemical method and observed that MLD increased together with the increase in Cox-2 expression. Consequently, the authors proposed that Cox-2 could be contributing to the formation of lymphatic vessels in esophageal cancer, thereby promoting metastasis. The present study revealed no correlation between Cox-2 expression and clinical factors in esophageal cancer. Cox-2 expression did not affect the DFS and OS curves of the patients and was not identified as a significant independent factor affecting survival rate, recurrence or metastasis of esophageal cancer. However, the OS and DFS curves were clustered according to Cox-2 expression, and the prognosis for the patients with negative Cox-2-expressing tumors was improved compared with patients with positive Cox-2-expressing tumors. Prins *et al* (25) noted that Cox-2 expression was associated with prognosis in esophageal adenocarcinoma and could be used as a risk stratification parameter in esophageal adenocarcinoma. However, in China, the more prevalent subtype of esophageal cancer is esophageal squamous cell carcinoma, and consequently, all cases included in the present study are of esophageal squamous carcinoma. The tumor subtype may account for the difference between the two studies.

P53/Cox-2 co-expression may have prognostic value in esophageal cancer. P53 and Cox-2 are expressed at higher levels in esophageal cancerous tissues compared with normal tissues. Mutations in the P53 gene are induced by various factors and lead to a loss of P53 tumor cell growth-inhibiting functions. Mutated P53 promotes tumor cell proliferation, inhibits apoptosis and promotes the occurrence and development of esophageal cancer. Although the specific mechanism remains unclear, Cox-2 acts as a cancer-promoting gene and plays a role in mediating angiogenesis, tumor growth, invasion and metastasis. This resulted in the hypothesis that there may be a synergistic association between Cox-2 and P53 in the occurrence and development of esophageal cancer. Benoit *et al* (26) reported that the P53 tumor suppressor gene could recruit nuclear factor (NF)-κB to transcriptionally activate Cox-2 expression and activity. Song *et al* (27) noted that blocking Cox-2 expression using small interfering RNA reinforced P53 transcriptional activity. Cheng *et al* (28) reported that specific Cox-2 inhibitors could completely reverse the inhibition of apoptosis induced by P53 and hepatitis virus X, suggesting that HBx could block P53-induced apoptosis through the Cox-2/prostaglandin E (2) signaling pathway. Choi *et al* (29) showed that Cox-2 expression reduced the expression of P53 and led to the inactivation of the P53 gene, thus promoting tumor development. Ma *et al* (30) observed that in precancerous lesions, tumor development is promoted via a cell survival mechanism by the interaction between Cox-2 and wild-type P53. However, in the late stage of tumor development, cells could resist apoptosis by relying on Cox-2 alone, without wild-type P53. This outcome may be due to the independence of the Cox-2 activation mechanism on P53 and NF-κB activity, or the occurrence of other cellular modifications to avoid apoptosis. Another mechanism may exist in lesions under inflammatory stress, where growth promoting signaling cascades (including Wnt/β-catenin, KRAS or c-Myb) activate the promoter and upregulate Cox-2 expression levels. Therefore, P53 and NF-κB action may not be the factors that activate Cox-2 expression. In addition, mutated P53 proteins may coexist with Cox-2 in the same cells and could synergize to inhibit cell apoptosis, thereby enhancing the malignant behavior of tumors and resulting in a significantly poorer prognosis. In the present study, P53 expression was observed in 60.5% (118/195), Cox-2 expression in 69.7% (136/195) and co-expression of P53 and Cox-2 in 43.1% (84/195) of the cases, with the expression of the two proteins being positively correlated. Using single factor Kaplan-Meier analysis, differences in survival rate between the P53/Cox-2 double-positive group compared with the other groups were identified. Furthermore, the two-year OS and DFS in the P53/Cox-2 double-positive group were significantly reduced compared with those in the other groups (75.0% vs. 85.6% and 63.9% vs. 81.8%, respectively) and have smaller P-values when compared with the group expressing P53 alone. Cox multivariate regression model analysis identified P53/Cox-2 co-expression as an independent factor influencing DFS and OS in esophageal carcinoma due to a larger HR and a smaller P-value compared with P53 expression alone. Analysis using a binary logistic regression model revealed that P53/Cox-2 co-expression also influenced the recurrence and metastasis of esophageal cancer, further implying that this may be used as a risk stratification parameter for the prognosis of esophageal cancer.

In conclusion, in the present study P53 and Cox-2 were markedly expressed in esophageal cancer tissues. P53 and Cox-2 co-expression was associated with increased malignant behavior of tumors and predicted a poor prognosis. Therefore, P53/Cox-2 co-expression may be used as a potential risk stratification parameter in esophageal cancer and may also be a promising therapeutic target. The pitfall of the present study lies in the short follow-up period following surgery, therefore, further complementary studies will aid in the thorough elucidation of the mechanisms behind P53 and Cox-2 interactions.

## Figures and Tables

**Table I.** The associations between P53 and Cox-2 expression and P53/Cox-2 co-expression and the assessed clinical factors.

	P53 expression			Cox-2 expression			P53 and Cox-2 co-expression		
						
Clinical factors	Total	High n=118	Low n=77	P-value	High n=136	Low n=59	P-value	P53(+) Cox-2(+) n=84	Other groups n=111	P-value
Age (years)				0.028^a^			0.641			0.056
<60	68	34	34		46	22		23	45	
≥60	127	84	43		90	37		61	66	
Gender				0.538			0.378			0.635
Female	45	29	16		29	16		18	27	
Male	150	89	61		107	43		66	84	
Differentiation				0.015^a^			0.200			0.020^a^
High	16	13	3		10	6		8	8	
Moderate	108	56	52		71	37		37	71	
Poor	71	49	22		55	16		39	32	
Position				0.078			0.285			0.560
Upper	7	7	0		3	4		3	4	
Middle	155	90	65		110	45		64	91	
Lower	33	21	12		23	10		17	16	
TNM stage				0.499			0.309			0.662
I	22	12	10		14	8		8	14
II	91	59	32		60	31		38	53
III	82	47	35		62	20		38	44

a P<0.05.

**Table II.** Cox multivariate analysis: The associations between clinical factors and esophageal cancer survival rates.

	Overall survival			Disease-free survival		
		
Characteristic	HR	95% CI	P-Value	HR	95% CI	P-value
Age (≥60 vs. <60 years)	1.129	0.613–2.077	0.697	1.157	0.628–2.130	0.641
Gender (female vs. male)	0.863	0.423–1.763	0.686	0.908	0.445–1.855	0.792
Position (upper vs. middle vs. lower)	1.329	0.690–2.560	0.396	1.319	0.686–2.538	0.406
Differentiation (high vs. moderate vs. poor)	1.254	0.796–1.974	0.329	1.252	0.795–1.971	0.332
TNM stage (I vs. II vs. III)	3.379	1.919–5.952	<0.001^a^	3.497	1.979–6.181	<0.001^a^
P53 expression (low vs. high)	2.102	1.108–3.991	0.023^a^	2.138	1.127–4.056	0.020^a^
Cox-2 expression (low vs. high)	1.473	0.742–2.923	0.268	1.453	0.734–2.875	0.283
P53(+) Cox-2(+) vs. other groups	2.212	1.219–4.012	0.009^a^	2.221	1.228–4.017	0.008^a^

a P<0.05. HR, hazard ratio; CI, confidence interval.

**Table III.** Binary logistic regression analysis: The associations between clinical factors and recurrence or metastasis in esophageal carcinoma.

	Recurrence or metastasis		
		
Characteristic	OR	95% CI	P-value
Age (≥60 vs. <60)	0.862	0.425–1.747	0.680
Gender (female vs. male)	1.432	0.609–3.371	0.411
Position (upper vs. middle vs. lower)	1.456	0.670–3.164	0.343
Differentiation (high vs. moderate vs. poor)	1.964	1.099–3.508	0.023^a^
TNM (I vs. II vs. III)	3.206	1.763–5.830	<0.001^a^
P53 expression (low vs. high)	2.510	1.228–5.131	0.012^a^
Cox-2 expression (low vs. high)	1.583	0.740–3.383	0.236
P53(+) Cox-2(+) vs. other groups	2.204	1.124–4.322	0.021^a^

a P<0.05. OR, odds ratio; CI, confidence interval.
